# Synergistic Interactions within a Multispecies Biofilm Enhance Individual Species Protection against Grazing by a Pelagic Protozoan

**DOI:** 10.3389/fmicb.2017.02649

**Published:** 2018-01-09

**Authors:** Prem K. Raghupathi, Wenzheng Liu, Koen Sabbe, Kurt Houf, Mette Burmølle, Søren J. Sørensen

**Affiliations:** ^1^Laboratory of Microbiology, Department of Veterinary Public Health and Food Safety, Faculty of Veterinary Medicine, Ghent University, Ghent, Belgium; ^2^Section for Microbiology, Department of Biology, University of Copenhagen, Copenhagen, Denmark; ^3^Laboratory of Protistology and Aquatic Ecology, Department of Biology, Faculty of Sciences, Ghent University, Ghent, Belgium

**Keywords:** synergy, multispecies biofilm, *Tetrahymena pyriformis*, grazing, species protection

## Abstract

Biofilm formation has been shown to confer protection against grazing, but little information is available on the effect of grazing on biofilm formation and protection in multispecies consortia. With most biofilms in nature being composed of multiple bacterial species, the interactions and dynamics of a multispecies bacterial biofilm subject to grazing by a pelagic protozoan predator were investigated. To this end, a mono and multispecies biofilms of four bacterial soil isolates, namely *Xanthomonas retroflexus, Stenotrophomonas rhizophila, Microbacterium oxydans* and *Paenibacillus amylolyticus*, were constructed and subjected to grazing by the ciliate *Tetrahymena pyriformis*. In monocultures, grazing strongly reduced planktonic cell numbers in *P. amylolyticus* and *S. rhizophila* and also *X. retroflexus*. At the same time, cell numbers in the underlying biofilms increased in *S. rhizophila* and *X. retroflexus*, but not in *P. amylolyticus*. This may be due to the fact that while grazing enhanced biofilm formation in the former two species, no biofilm was formed by *P. amylolyticus* in monoculture, either with or without grazing. In four-species biofilms, biofilm formation was higher than in the best monoculture, a strong biodiversity effect that was even more pronounced in the presence of grazing. While cell numbers of *X. retroflexus, S. rhizophila*, and *P. amylolyticus* in the planktonic fraction were greatly reduced in the presence of grazers, cell numbers of all three species strongly increased in the biofilm. Our results show that synergistic interactions between the four-species were important to induce biofilm formation, and suggest that bacterial members that produce more biofilm when exposed to the grazer not only protect themselves but also supported other members which are sensitive to grazing, thereby providing a “shared grazing protection” within the four-species biofilm model. Hence, complex interactions shape the dynamics of the biofilm and enhance overall community fitness under stressful conditions such as grazing. These emerging inter- and intra-species interactions could play a vital role in biofilm dynamics in natural environments like soil or aquatic systems.

## Introduction

In recent years, protozoa–bacteria interactions have received increasing attention in studies ranging from ecology to consumer health and diseases. Free-living protozoa are commonly found in natural environments like soils and aquatic habitats (Foissner, 1999; Ekelund et al., 2001; Pfister et al., 2002; Pernthaler, 2005) and in anthropogenic environments like swimming pools (Rivera et al., 1993), drinking water systems (Thomas and Ashbolt, 2011), kitchens (Chavatte et al., 2014) and health care facilities (Singh and Coogan, 2005; Cateau et al., 2014). Various studies have also reported the presence of bacterial biofilms in such environments (Bryers, 2008; Burmølle et al., 2011; Besemer et al., 2012). Though most studies emphasize that the main role played by the protozoa lies in control of the bacterial populations by predation (Jürgens and Güde, 1994; Brown and Barker, 1999; Arndt et al., 2003; Logares et al., 2012), another potential impact involves the induction of biofilm formation by bacterial communities (Joubert et al., 2006) to avoid grazing.

Biofilm formation represents a surface attached mode of life (Donlan, 2002) that can contain multiple species of archaea, bacteria, fungi, and algae (Flemming et al., 2016). Biofilms offer physical protection through the secreted polymeric matrix (Joubert et al., 2006) that creates a protective microhabitat against predation (Darby et al., 2002; Matz et al., 2005; DePas et al., 2014). Close interactions between bacteria and protozoa in biofilms are also thought to give rise to a series of adaptations in bacterial communities by promoting horizontal gene transfer events, quorum sensing abilities and induce bacterial protein secretion systems (Darby et al., 2002; Matz et al., 2004) enhancing their survival, dynamics and coexistence (Matz and Kjelleberg, 2005).

Grazing by protozoa has been reported to stimulate micro-colony formation, alter mass transfer of nutrients and induce biofilm development by stimulating bacterial layer thickness (Matz et al., 2004; Weitere et al., 2005; Kaminskaya et al., 2007; Wey et al., 2008; Böhme et al., 2009). Other studies, however, argue that protozoa do not induce biofilm formation (Huws et al., 2005) but instead show a marked preference for grazing on attached or aggregated bacterial cells or only change biofilm community structure (Caron, 1987; Sibbald and Albright, 1988; Huws et al., 2005; Wey et al., 2008). Furthermore, studies have also shown that the grazed or consumed bacterial cells can become adapted to resist uptake or digestion and are even capable of intracellular replication within the protozoan host cells (Rowe and Grant, 2006; Taylor et al., 2009; Lambrecht et al., 2015). Although feeding interactions between protozoa and planktonic bacteria are well understood (Jürgens and Matz, 2002; Matz and Jürgens, 2005; Roberts et al., 2011) only few studies have attempted to assess the impact of grazing on biofilms at the multi-species level. In multispecies biofilm settings, interactions between different bacteria play an important role in determining the structure, function and dynamics of the biofilms and it has been suggested that they contribute to defense mechanisms of bacterial biofilms against predators (Wey et al., 2008; Matz, 2011). Moreover, it has been shown that interspecific interactions within the mixed bacterial communities in the presence of a grazing protist promoted co-aggregation of bacterial members and enhanced complex biopolymer degradation pathways leading to an overall increase in carbon transfer efficiency (Corno et al., 2013, 2015). Mixed biofilms have in other cases been shown to offer the harbored species protection against antibacterial compounds and enhanced capabilities of invasion and virulence within host organisms (Burmølle et al., 2014).

Different protozoan members have different impact on the microbial communities (Brown and Barker, 1999; Paisie et al., 2014). In soils, protozoa present themselves as a diverse group of flagellates, ciliates, and naked amoebae (Ekelund and Rønn, 1994; Bonnet et al., 2005). Like flagellates, ciliates display a substantial diversity in motility, morphology and feeding strategies (Dopheide et al., 2011) and are considered to be important predators of bacteria. Hence, there is a need to unravel different prey-predator interactions and their impact on mixed species bacterial biofilm, as mixed biofilms are the predominant lifestyle in most ecosystems (Battin et al., 2003; Costerton, 2007; Mielich-Süss and Lopez, 2015). Grazing on diverse biofilms is likely to shape the existing complex interactions within the biofilm communities (Wen et al., 2010; Hansen et al., 2017) or alter the feeding traits of protozoa. Examples include Gram-negative bacteria being more vulnerable to grazing than Gram-positive bacteria (Rønn et al., 2002) or altered feeding responses of protozoa to one bacterial group over another (Dopheide et al., 2011).

The aim of the present study is to assess whether individual biofilm bacterial species gain enhanced protection by other members in multispecies consortia under grazing pressure. Therefore, we examined the effect of grazing by the ciliate *Tetrahymena pyriformis* on biofilm formation and population dynamics in a consortium composed of four bacterial soil species *Xanthomonas retroflexus, Stenotrophomonas rhizophila, Microbacterium oxydans*, and *Paenibacillus amylolyticus*. These four strains when combined have been shown to act synergistically resulting in increased biofilm development (Ren et al., 2015). Ciliates were shown to be effective bacterial grazers with often extremely high ingestion rates (Iriberri et al., 1995), making them a specialized subgroup within the protist (Parry, 2004). Under such extreme grazing pressure, we hypothesize that multispecies biofilms will generate a protective effect compared to single species biofilms. We used a qPCR protocol developed previously for these model consortia (Ren et al., 2014) to quantify the species-specific impact of protozoan grazing.

## Materials and Methods

### Soil Isolates and Protozoa Culture Conditions

The bacterial species *X. retroflexus* (*JQ890537*), *S. rhizophila* (*JQ890538*), *M. oxydans* (*JQ890539*), and *P. amylolyticus* (*JQ890540*) stored in the culture collection of the Section of Microbiology, University of Copenhagen, were subcultured from frozen glycerol stocks onto TSA plates (Tryptic Soy Agar, Sigma–Aldrich, Germany). The plates were incubated at 24°C for 48 h. Single colonies were inoculated into 5 ml TSB (Tryptic Soy Broth, Sigma–Aldrich, Germany) media and incubated with shaking at 180 rpm for 24 h at 24°C when required. These strains were used as bacterial prey for the protozoan predator.

A *T. pyriformis* (Tp) culture (Culture Collection of Algae and Protozoa, CCAP nr 1630/w1) was provided by Department of Veterinary Public Health and Food Safety, Ghent University, Belgium. Axenic cultures of this protozoan were maintained in 25 cm^2^ culture flasks with 20 ml PPY medium [proteose peptone yeast extract; 20 g Proteose peptone (Merck KgaAm Germany), 2.5 g yeast extract (Merck KgaAm Germany) in 1 L H_2_O; autoclaved]. Weekly maintenance of the ciliate cultures at 24°C was done by aseptically transferring 5 ml of the culture into 15 ml fresh PPY medium incubated. For biofilm grazing experiments, *T. pyriformis* cells in exponential phase (after 48 h at 24°C) were washed twice in PAS (Page’s amoeba saline) solution followed by centrifuging at 850 g after which the cells were re-suspended into 10 ml TSB media.

### Biofilm Cultivation and Grazing Experiments

Biofilm cultivation experiments were performed in 96-well cell culture plates (cat. no. 655180, Greiner Bio One, Germany). The four selected strains were screened for biofilm formation as single species (monospecies) and in three/four-species combination (multispecies) as described (Ren et al., 2015) both in the presence and absence of protozoa. Briefly, bacterial cell cultures in exponential growth phase (OD_600_ between 0.3 – 0.6) were selected and adjusted to a start OD_600_ of 0.15 in TSB media for all cultures. For monospecies biofilms, aliquots of 150 μl of cell culture and for three- and four-species biofilms, respectively, 50 or 37.5 μl of each bacterial strain were added into the wells so that the final inocula were 150 μl in all the settings. To the wells that were to be grazed, an volume of 1.5 μl containing ∼approximately 1000 cells *T. pyriformis* cells in TSB media were added. The plates were incubated at 24°C for 12, 24, and 96 h. Wells containing only 150 μl TSB media and TSB media with *T. pyriformis* cells served as blank/control. Three wells each time served as one technical replicate and this was repeated at five different times.

### Quantification of Biofilm and Planktonic Fractions

Biofilm formation was assayed and quantified using the traditional crystal violet (CV) method as previously described (Ren et al., 2014). The biofilm attached to the wells was then washed twice gently with 160 μl 1X PBS (phosphate buffer saline) solution and stained with 180 μl 1% (w/v) CV solution. After 20 min of staining, the CV solution was removed by pipette, and the stained biofilm was gently washed five times with 200 μl PBS solution. The remaining CV dye retained by the biofilm was de-stained into 200 μl 96% ethanol for 30 min. Biofilm formation was then quantified by measuring the absorbance of de-stained CV at 590 nm using EL340 BioKinetics reader (BioTek Instruments, United States) and expressed as biofilm forming index (BFI) according to the equation BFI = (AB-CW)/G (Niu and Gilbert, 2004) where, AB: OD_590_ of attached microorganisms, CW: OD_590_ control wells and G: OD_600_ of cells in planktonic fraction. Biodiversity (BD) effect was calculated as the difference between the observed biofilm yield (biofilm of mixed cultures) and the expected yield (average of the monoculture yields) (Loreau and Hector, 2001; Vanelslander et al., 2009). Biofilm fold (*F*_d_), i.e., the observed increase in biofilm formation due to grazing is the ratio between OD_590_ of grazed three-species biofilm and OD_590_ of non-grazed three-species biofilm.

Quantification of the biofilm and planktonic fractions was performed by plating. 100 μl of the planktonic fraction from the wells after 24 and 96 h incubation was suspended in 900 μl 1X PBS solution. Once the planktonic fractions were removed, the wells with attached biofilm were gently washed twice with 160 μl 1X PBS solution. The wells were then filled with 200 μl 1X PBS and mixed thoroughly by pipetting. Serial dilutions in 900 μl 1X PBS were performed and 100 μl of the dilutions were plated onto TSA plates by spread plating after which the plates were allowed to dry completely at room temperature. Drying restricts the movement of *T. pyriformis* on plates. The plates were then incubated for 48 h at 24°C. Single colonies formed after incubation were counted and the results were calculated in CFU (colony forming units). Grazing fold, i.e., the percentage reduction in planktonic fraction due to grazing was expressed by 100 × [CFU_(culture)_ - CFU_(culture+Tp)_/CFU_(culture)_]. Changes in cell counts from biofilm fraction were expressed by log [CFU_(culture+Tp)_/CFU_(culture)_].

### Ciliate Growth on Bacterial Cultures

We determined the ciliate numbers of *T. pyriformis* grown on the four bacteria separately (monospecies) and as a mixture (four-species) for up to 96 h at regular intervals in microtiter plates. The protozoa cells were counted using a Sedgewick-Rafter chamber and an inverted microscope (40× magnification) as described previously (Gittleson and Ganapathy, 2011) with minor modifications. The wells containing the suspension of bacteria and protozoa were homogenized by pipetting and 150 μl of the cell suspension was fixed in 1% (w/v) Lugol’s iodine solution to a final volume of 1.2 ml in dH_2_O. The contents were then immediately transferred to the counting chamber and the cells were allowed to settle for few minutes. The change in protozoa cell numbers over time was expressed using Δ*N* = log_10_ (*N*_t_ -*N*_0_)/*t*. To visualize the changes in protozoa numbers over time in co-culture with bacteria, 50 μl spots of the fixed suspension were made on glass slides and micrographs were taken at different time points using Zeiss Axioplan II, Carl Zeiss with a 10× objective.

### 16S rRNA Based Fluorescent *in Situ* Hybridization (FISH) and Confocal Imaging to Investigate Grazing

To visualize the effects of grazing by the protozoan and the internalization of bacteria within the food vacuoles of *T. pyriformis*, FISH was performed with 16S rRNA gene probes targeting the specific bacteria (Liu et al., 2017). 50 μl spots of co-culture suspensions (bacteria and protozoa) after 24 h were collected after thorough pipetting to homogenize the suspension. The collected cells were then left to air dry on a glass slide. The above step was repeated five times (5 μl × 50 μl) with the aim to collect more cells. The attached cells were coated with 0.5% (w/v) agarose by immersing the slides into a tube containing 45 ml molten agarose and fixed using 4% PFA (paraformaldehyde) at 4°C. Samples were dehydrated and the hybridization protocol was performed according to (Amann, 1995; Daims, 2009) with 30% formamide concentration. After hybridization, the slides were washed in cold water and dried at room temperature. The slides were stored in the dark and visualized under confocal microscopy (Point-scanning confocal and multiphoton microscope SP5-X MP, Leica Microsystems). Images were processed using Leica Application Suite X.

### *qPCR* Quantification of Bacterial Cell Numbers in Multispecies Setting

The biofilm formation assay was conducted both in the presence and absence of *T. pyriformis* in 96-well microtiter plates as described above. After 24 h, the planktonic fractions were collected in Eppendorf tubes and the biofilm fraction was rinsed twice with weak phosphate buffer to remove loosely attached cells. Three replicate wells were prepared for each treatment. The cell numbers of the four strains in multispecies planktonic and biofilm fractions with and without protozoa were quantified by SYBR Green qPCR using standard curves generated by serial 10-fold dilutions of plasmid DNA using the species specific primers and thermal profile setup previously reported (Ren et al., 2014). All samples were run in triplicate and a no template control was included in each run. Bacterial DNA was extracted using FastDNA^TM^ SPIN Kit for soil (MP Biomedicals, Germany) according to manufacturer’s instruction.

## Results

### *T. pyriformis* Grazing Promotes Biofilm Formation and Reduces the Number of Bacteria in the Planktonic Fractions

**FIGURE 1:**
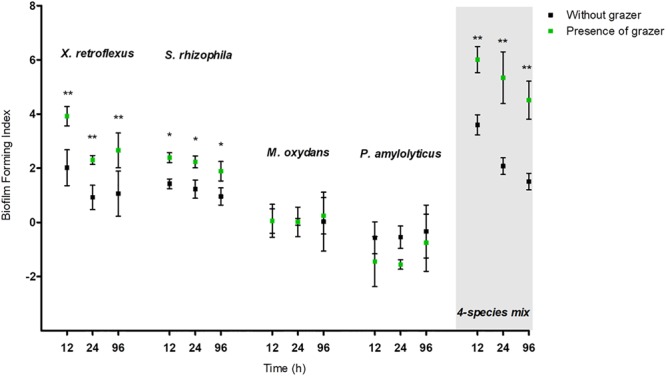
Biofilm forming index (BFI) of mono and mixed species cultures subject to *Tetrahymena pyriformis* (Tp) grazing and non-grazed cultures at 12, 24, and 96 h. The data points indicate the biofilm mean ± standard error of the mean (SEM) obtained from five biological replicates.^∗^*P* < 0.05, ^∗∗^*P* < 0.01.

Monocultures and four-species mixed cultures of *X. retroflexus, S. rhizophila, M. oxydans*, and *P. amylolyticus* were tested for biofilm formation in the absence and presence of protozoa (**Figure 1**). *T. pyriformis* grazing on monospecies cultures of *X. retroflexus* and *S. rhizophila* resulted in significantly enhanced biofilm formation (paired *t*-test, *P* < 0.05) whereas *M. oxydans* and *P. amylolyticus* monocultures did not form biofilms neither in the presence nor in the absence of *T. pyriformis*. Biofilm formation was enhanced in the four-species mixtures, and was even more strongly induced in these mixtures in the presence of grazing for up to 96 h (*n* = 5, paired *t*-test, *P* < 0.05), suggesting a strong biodiversity effect (Supplementary Figure S1). Moreover, biofilm formation in the mixtures was higher than in the best performing monoculture both in the absence and presence of grazing.

**FIGURE 2:**
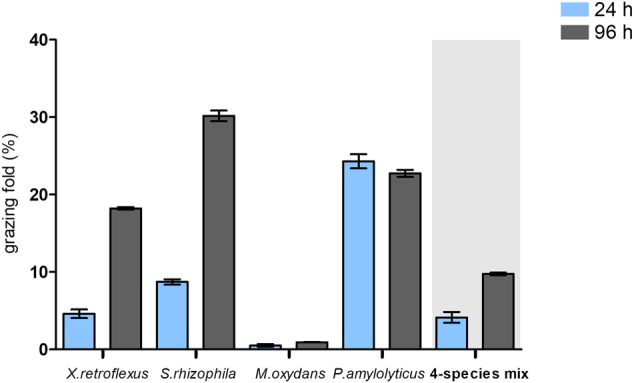
Percentage of mono and multispecies planktonic cultures grazed by *T. pyriformis* after 24 and 96 h compared to the non-grazed cultures. The data points indicate the percentage reduction in cell numbers (%) ± SEM obtained from five biological replicates.

Planktonic fractions of three of the four bacterial species were effectively grazed upon in monoculture, but less so in the four-species co-culture. *P. amylolyticus* was the most intensively grazed species at 24 h, whereas after 96 h *S. rhizophila* monocultures were the most highly grazed followed by *P. amylolyticus* and *X. retroflexus* monocultures. Among all the strains, *M. oxydans* was the least preferred prey, and *S. rhizophila* and *P. amylolyticus* were the most favored prey (**Figure 2**). These grazing experiments verified the ability of *T. pyriformis* to feed on planktonic bacteria. In the four-species mixed cultures, overall grazing by *T. pyriformis* on the planktonic community was reduced compared to the monospecies cultures observed by the low grazing fold values at 24 and 96 h (**Figure 2**).

**FIGURE 3:**
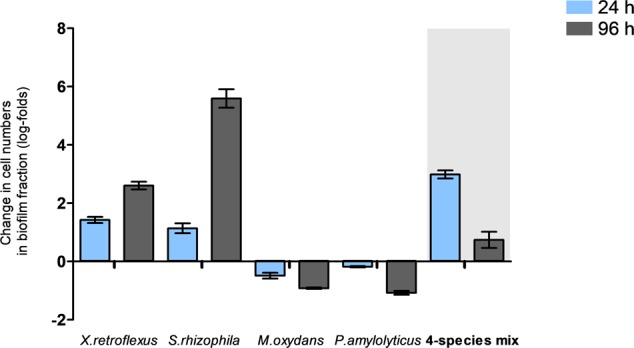
Change in viable cell numbers from the biofilm fractions of mono and multispecies cultures grazed with *T. pyriformis* after 24 and 96 h obtained from plating. The data points indicate the change in cell numbers of grazed biofilm fraction relative to the non-grazed biofilm fraction ± SEM obtained from two biological replicates.

In the biofilm fraction, cell numbers of *X. retroflexus* and *S. rhizophila* increased at 24 and 96 h in the grazed relative to the non-grazed monocultures whereas the cell numbers of *M. oxydans* and *P. amylolyticus* decreased with grazing compared to the non-grazed monocultures (**Figure 3**). This underscores the inability of *M. oxydans* and *P. amylolyticus* to form a biofilm in monoculture. In the four-species culture, total cell numbers increased both at 24 and 96 h compared to the non-grazed biofilm (**Figure 3** and Supplementary Figure S2).

### Growth of *T. pyriformis* on Bacterial Cultures

**FIGURE 4:**
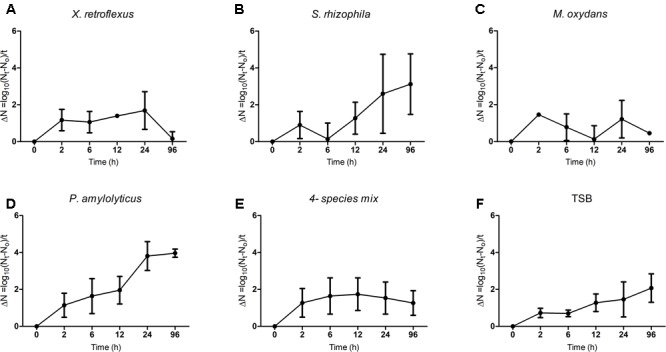
*Tetrahymena pyriformis* growth curves. The data points indicate the change in protozoan cell numbers with respect to time (*t* = 0 h) that were grown in co-culture with the bacterial isolates **(A–D)** as monocultures and **(E)** as four-species mixed culture. **(F)** Change in protozoan cell numbers under axenic conditions over time in TSB media. The data shown are mean ± SEM from three biological replicates.

The growth of *T. pyriformis* cells on all bacterial isolates cultured as both mono and mixed planktonic cultures was followed over time (**Figure 4**). The change in cell numbers over time demonstrated that *S. rhizophila* and *P. amylolyticus* were suitable prey for the protozoa (**Figures 4B,D**) and that TSB media can support the axenic growth of protozoa (**Figure 4F**). Growth on *M. oxydans* was not pronounced (**Figure 4C**); while *X. retroflexus* monocultures had a negative impact on the growth of the protozoa at 96 h (**Figure 4A**). Our results thus indicate that *T. pyriformis* may prefer to graze on *S. rhizophila* and *P. amylolyticus*. The numbers of protozoa grazing on the four-species mixed cultures represent a smoother curve over time indicating that the protozoa can adapt to an available prey in multispecies bacterial environments (**Figure 4E**).

To visualize the change in protozoan numbers over time, micrographs showing *T. pyriformis* cells raised on both mono and multispecies bacterial cultures are shown (Supplementary Figure S3). The protozoan population raised on the four-species mixtures remained viable for up to 96 h. However, in monospecies cultures; it can be seen that the protozoan cell numbers increased from 24 h and reached a maximum at 96 h when co-cultured with *S. rhizophila* and *P. amylolyticus* whereas the protozoa population declined from 24 to 96 h in co-culture with *X. retroflexus* and *M. oxydans*.

### Grazed Bacterial Prey within the Food Vacuoles of *T. pyriformis*

**FIGURE 5:**
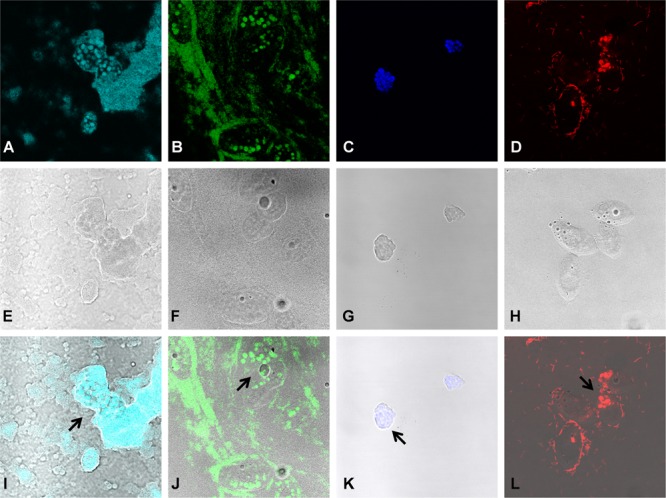
Grazing by protozoa on monospecies bacterial cultures. FISH based staining and confocal imaging shows *Xanthomonas retroflexus* (FL: **A**, BF: **E**, and OL: **I**), *Stenotrophomonas rhizophila* (FL: **B**, BF: **F**, and OL: **J**), *Microbacterium oxydans* (FL: **C**, BF: **G**, and OL: **K**) and *Paenibacillus amylolyticus* (FL: **D**, BF: **H**, and OL: **L**) cells, cultured as monospecies, localized within the food vacuoles (indicated by the arrows) of *T. pyriformis* cells after 24 h of grazing. ‘FL’ denotes fluorescence, ‘BF’ denotes bright-field and ‘OL’ denotes overlay images, respectively.

**FIGURE 6:**
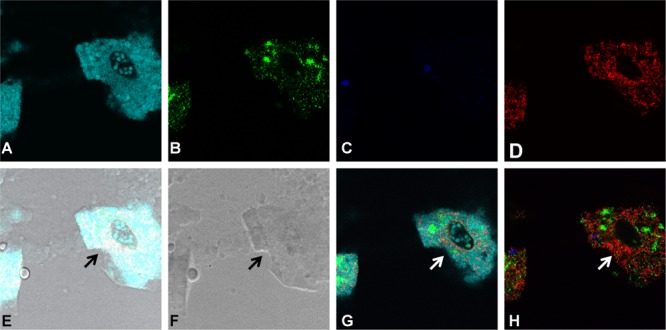
Grazing for 24 h by protozoa on the four-species mixed cultures. FISH based staining and confocal imaging shows the distribution of the different bacterial species in and around the protozoan cells. Applying the fluorescence filter channels, it is observed that *X. retroflexus* is abundantly present within the food vacuoles (indicated by the arrows) of *T. pyriformis* **(A)**. *S. rhizophila* **(B)** is detected to a lesser extent whereas *M. oxydans* **(C)** and *P. amylolyticus* **(D)** cells are not visibly present in the food vacuoles. **(E,F)** Depict the overlay and bright-field images, respectively. Panels **(G,H)** were included to phase out the dominating fluorescence signals from *X. retroflexus* and visualize the other bacterial members in the biofilm consortia around the ciliate.

To visualize grazing on monocultures and mixed cultures, a 16S rRNA gene based FISH was performed, similar to a previous study (Jezbera et al., 2005), after 24 h of grazing and samples were visualized by laser scanning confocal microscopy. It was confirmed that *T. pyriformis* can consume the bacteria in all tested monospecies settings, however, at seemingly different rates as indicated by the number of food vacuoles formed within the ciliates (**Figure 5**). In co-cultures of *T. pyriformis* with *X. retroflexus* or *S. rhizophila* monocultures, the bacteria were abundantly present within the food vacuoles of *T. pyriformis* cells (**Figures 5A,B**) showing that these bacterial strains are readily consumed. *P. amylolyticus* cells were also found to be localized within the food vacuoles of grazers but not as abundantly as compared to *X. retroflexus* and *S. rhizophila* (**Figure 5D**). Most protozoan cells appeared to form cysts when co-cultured with *M. oxydans* (**Figure 5C**), but some bacterial cells were found to be internalized within *T. pyriformis* indicating that the protozoa were able to consume *M. oxydans* cells. In the case of grazing on four-species mixed cultures (**Figures 6A–H**), most food vacuoles were dominated by *X. retroflexus* indicating that at 24 h most protozoan cells prefer to graze on *X. retroflexus.* This was in accordance with the fact that this bacterium previously was shown to dominate the 24 h mixed biofilm population (Ren et al., 2014) and thus could be readily available for the grazers.

### Biofilm Formation by *X. retroflexus* Is Vital to the Overall Biofilm Development

**FIGURE 7:**
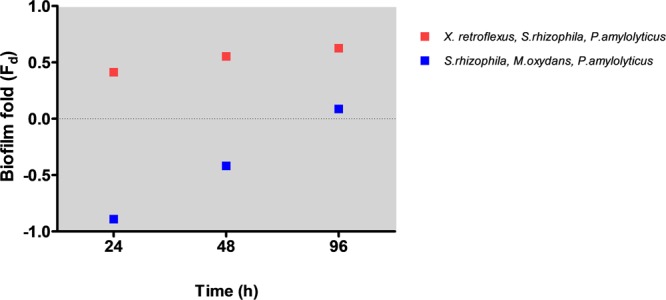
Biofilm formation in the presence of *T. pyriformis* in three-species bacterial consortia. *X. retroflexus* is vital for biofilm development. Biofilm fold was calculated as the ratio of Abs_590_ [(three – species biofilm cultured with grazer cells + SEM) – (three – species biofilm as control – SEM)] to Abs_590_ (three – species biofilm cultured with grazer cells + SEM).

From the above results, biofilm formation in the presence of *T. pyriformis* was further assessed to better understand the dynamics. To this end, either the least preferred prey *M. oxydans* or the best biofilm producer *X. retroflexus* were excluded three-species consortia (**Figure 7**). Biofilm formation (biofilm-fold *F*_d_) was enhanced when *X. retroflexus* remained in the consortium together with *S. rhizophila* and *P. amylolyticus*, indicating that the interaction between these three members is vital for biofilm stability. However, in the absence of *X. retroflexus* and in the presence of *M. oxydans*, the consortium was effectively grazed, although there seemed for this consortium to be a gradual adaptation to predation (as evidenced by increased biofilm formation) over time.

### Impact of Grazing on the Population Dynamics of Individual Bacterial Species in Multispecies Biofilm and Planktonic Consortia

**FIGURE 8:**
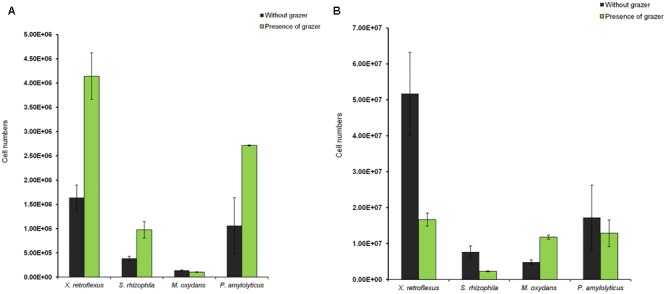
Impact of grazing by *T. pyriformis* on the population dynamics of the individual bacterial species in the multispecies consortia as assessed by qPCR. Cell numbers of the individual bacterial members in **(A)** the multispecies biofilm fraction and **(B)** the multispecies planktonic fraction after 24 h of grazing.

In order to determine the cell numbers of the individual species within the multispecies consortium, 16S rRNA gene based q-PCR quantification was applied according to a previously developed protocol (Ren et al., 2014). The results showed that in the multispecies biofilm fraction, cell numbers of *X. retroflexus, S. rhizophila*, and *P. amylolyticus* increased in the presence of grazers compared to the control biofilms that were not grazed. The ∼2.5-fold increase in cell numbers of *X. retroflexus* and *P. amylolyticus* and 1.7-fold increase in *S. rhizophila* cell numbers suggest that synergistic interactions between these species were enhanced in the presence of grazing, resulting in increased cell numbers in the biofilm. The cell numbers of *M. oxydans* in the biofilm remained unaffected either in the presence or absence of grazers (**Figure 8A**).

In the planktonic fraction without grazing, a similar trend in cell numbers compared to the non-grazed biofilm was seen with *X. retroflexus, P. amylolyticus*, and *S. rhizophila* being the dominant species (**Figure 8B**). However, the planktonic cell numbers of these species decreased in the presence of *T. pyriformis* indicating an effect of grazing on these planktonic fractions. In contrast, the cell numbers of *M. oxydans* increased, which possibly can be a result of grazing preference of the ciliate in the mixed communities and/or higher nutrient or space availability for *M. oxydans* cells as the other members of the consortia were grazed upon.

## Discussion

In the present study, the impact of grazing by the ciliate *T. pyriformis* on a previously described synergistic mixed species biofilm model consortium (Ren et al., 2015) was assessed. These bacterial strains were isolated from a single micro-habitat and studies have reported that long-term coexistence within a habitat can stimulate synergistic biofilm development in complex communities (Madsen et al., 2016). Our results showed that co-culturing *T. pyriformis* with single-species bacterial cultures stimulated biofilm formation in *X. retroflexus* and *S. rhizophila* strains but not in *M. oxydans* and *P. amylolyticus* (**Figure 1**). *S. rhizophila* and *P. amylolyticus* were most sensitive to grazing (**Figure 2**). Ciliate abundances reached a maximum in co-culture with these strains over time indicating extensive feeding on these strains (**Figures 4B,D** and Supplementary Figure S1). The monospecies grazing experiments thus indicate differential bacterial behavior in response to a predator and vice-versa. Similar observations have been reported previously where protozoa regulate the social behavior of the bacteria (Rønn et al., 2002; Scherwass et al., 2016) or where bacteria regulate the protozoan population (Kaminskaya et al., 2007). The specificity of such responses has been reported to vary depending on the selected bacteria and protozoa (Dopheide et al., 2011; Friman et al., 2013).

In the four-species consortia, biofilm formation was enhanced even when compared to the best performing monoculture, suggesting a strong and significant biodiversity effect which was even further enhanced in the presence of grazing (**Figure 1** and Supplementary Figure S1). The total cell numbers in mixed biofilm fraction under grazed conditions were increased compared to non-grazed mixed biofilm (**Figure 3**) and at the same time, qPCR results showed that the bacterial numbers of all strains except *M. oxydans* increased in comparison with the non-grazed biofilm. Even the grazing sensitive species *P. amylolyticus* increased in cell numbers in the mixed biofilm during grazing. *X. retroflexus* dominated the grazed biofilm followed by *P. amylolyticus* and *S. rhizophila*, respectively (**Figure 8A**). This suggests strong synergistic and complex interactions between these species under grazing pressure, resulting in a shared protection against grazing. In contrast, total bacterial numbers in the multispecies planktonic fraction under grazing were reduced for all species, except *M. oxydans* (**Figure 8B**). This can be explained by the lowest grazing preference for *M. oxydans* in monoculture. Protozoan cell numbers in the mixed planktonic cultures (**Figure 4E**) gradually decreased with time, possibly reflecting a lower availability of the preferred individual prey or co-aggregation of the bacterial consortia members into composite aggregates.

In the mixed-species consortia there was an increase by ∼2.5-fold in total bacterial cell numbers (all four species combined) in the grazed biofilm compared to the non-grazed biofilm, whereas in the planktonic fractions grazing reduced total cell numbers by ∼1.8-fold, emphasizing the protective nature of the biofilm mode of life. Evidence that grazing pressure is positively correlated with the formation of cell clusters has come from both monospecies laboratory biofilm (Matz et al., 2004, 2005) and from natural/semi-natural multispecies biofilm (Wey et al., 2008; Rychert and Neu, 2010; Corno et al., 2015). Grazing induced biofilm formation could reflect either an active defense mechanism (Matz and Kjelleberg, 2005; Friman and Buckling, 2014) or a passive mechanical process where the movement of the protozoan cells drives the bacterial cells to the substratum (Wey et al., 2012). Also, protozoan grazing on the planktonic bacterial population could release nutrients which stimulate the biofilm-associated cells resulting in enhanced levels of biofilm formation (Petropoulos and Gilbride, 2005; Böhme et al., 2009). Additionally, the total bacterial productivity is shown to be influenced under grazing where bacterial aggregates display increased carbon transfer and uptake (Corno et al., 2013, 2015). Discrepancies found in the literature with respect to the protective nature of biofilms against grazing (Jackson and Jones, 1991; Huws et al., 2005; Weitere et al., 2005) could be attributed to the type of protozoa used, their feeding mechanism and the growth conditions. Studies have shown feeding traits of grazers to influence grazing resistance in bacterial biofilms (Seiler et al., 2017) and surface associated bacteria can be even more consumed when exposed to a specialized grazer (Rogerson and Laybourn-Parry, 1992). Therefore, more studies with different gazers are needed for a comprehensive understanding on the effect of grazing by protozoan on bacterial biofilm. In this study, we determined the grazing effect on a four species biofilm using a single pelagic grazer, the precise mechanisms that confer grazing resistance to individual species remains unknown. However, the biofilm formation was enhanced in a more diverse biofilm composed of four species, beyond the expected biofilm forming capacities of all monocultures, especially under grazed conditions. Thus, in a multispecies biofilm, the observed protection due to biofilm formation could be seen as a result of synergistic interactions or complementarity within the mixed cultures.

In addition, *X. retroflexus* dominated the multispecies biofilm while *M. oxydans* was the least preferred prey in monoculture. However, both these species have been shown to confer synergy and shared protection (Ren et al., 2015; Hansen et al., 2017; Liu et al., 2017). Different three-species co-cultures, set up to investigate the role of these two bacteria in the communal protection observed in the multispecies biofilm, showed that biofilm formation was enhanced by 3.5-folds in the three-species biofilm composed of *X. retroflexus, S. rhizophila*, and *P. amylolyticus* in the presence of grazers; but that the synergy was hampered when *X. retroflexus* was substituted by *M. oxydans* (**Figure 7**). From these results, it can be deduced that the intricate interactions between *X. retroflexus* and the other two members is vital for enhanced biofilm formation and communal grazing resistance. Grazing-sensitive members (*S. rhizophila* and *P. amylolyticus*) are more susceptible to grazing in the absence of key biofilm producers such as *X. retroflexus*. These results demonstrate that synergistic interactions within the multispecies communities are further enhanced under grazing pressure, as also observed by (Corno et al., 2015), and the multispecies biofilm architecture provided grazing sensitive members with improved protection (Burmølle et al., 2014). This emergent property of multispecies biofilms could serve as a public goods strategy, as previously reported for antimicrobials (Lee et al., 2014), and can thus act as a major driver for synergistic cooperative behavior.

Our findings support previous findings (Ren et al., 2015; Madsen et al., 2016) that bacteria can increase their fitness by engaging in the formation of multispecies biofilms. We showed that in multispecies consortium under grazing pressure, cell numbers of free floating bacteria decrease while biofilm cell numbers increase. Our findings thus suggest that synergy in biofilm formation could have evolved from the selective pressures under stressful environmental conditions such as grazing.

## Author Contributions

PR, SS, MB, and KH designed the study. PR performed the experiments. PR and WL analyzed the data. PR, WL, KS, KH, MB, and SS revised the manuscript. KH, KS, MB, and SS provided the final approval to publish.

## Conflict of Interest Statement

The authors declare that the research was conducted in the absence of any commercial or financial relationships that could be construed as a potential conflict of interest.
