# Supplementary material for: Synergistic Interactions within a Multispecies Biofilm Enhance Individual Species Protection against Grazing by a Pelagic Protozoan
Source: Front Microbiol. 2018 Jan 9;8:2649. doi: 10.3389/fmicb.2017.02649 (PMC5767253; doi:10.3389/fmicb.2017.02649)

Supplementary information

Figure S1: Net biodiversity effect calculated as the difference between the biofilm formation of the mixed culture and the biofilm yield expected on the basis of the average of the monocultures at 12, 24 and 96 hours. The biodiversity effect significantly increased in multispecies biofilm compared to the monocultures and was even more pronounced in the presence of protozoa (ANOVA, *P < 0.05). The data point indicates the mean BD ± SEM obtained from five biological replicates.

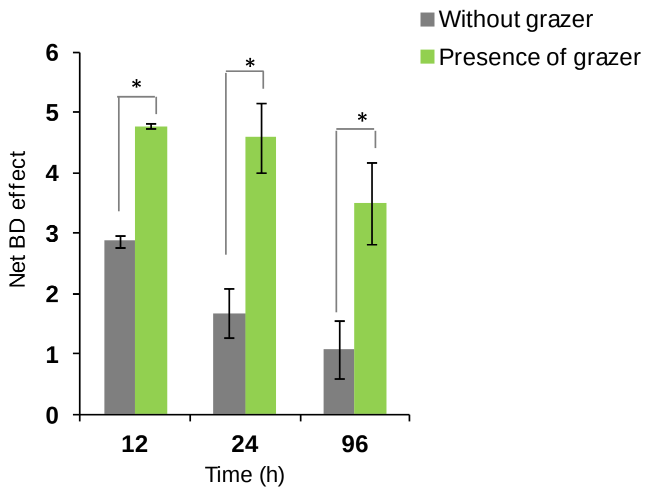

Figure S2: Quantification of **(A)** biofilm and **(B)** planktonic fractions of mono and mixed-species cultures in the presence and absence of grazing *Tetrahymena pyriformis* (Tp) after 24 and 96 hours obtained by plating. The data points indicate the mean colony forming unit (CFU) ± SEM obtained from two biological replicates.

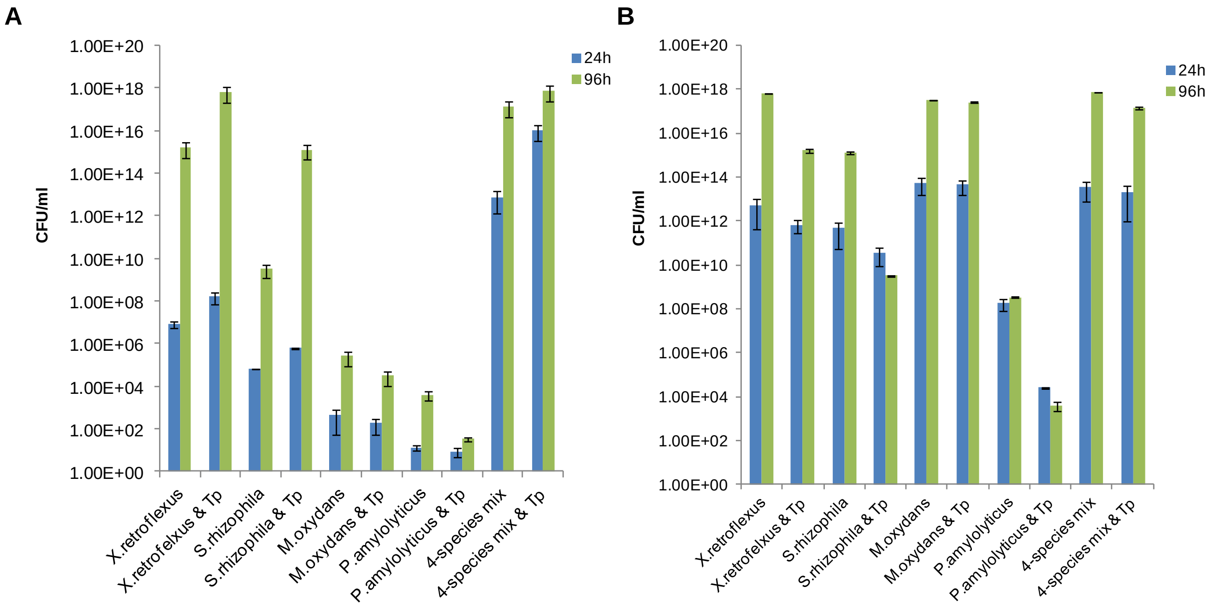

Figure S3: Micrographs depicting *T. pyriformis* cells in co-culture with monospecies, mixed-species bacterial cultures and in TSB media at 0.5 h, 6 h, 12 h, 24 h and 96 h. The protozoan-bacterial suspensions were fixed using 1% (w/v) Lugol's iodine solution.

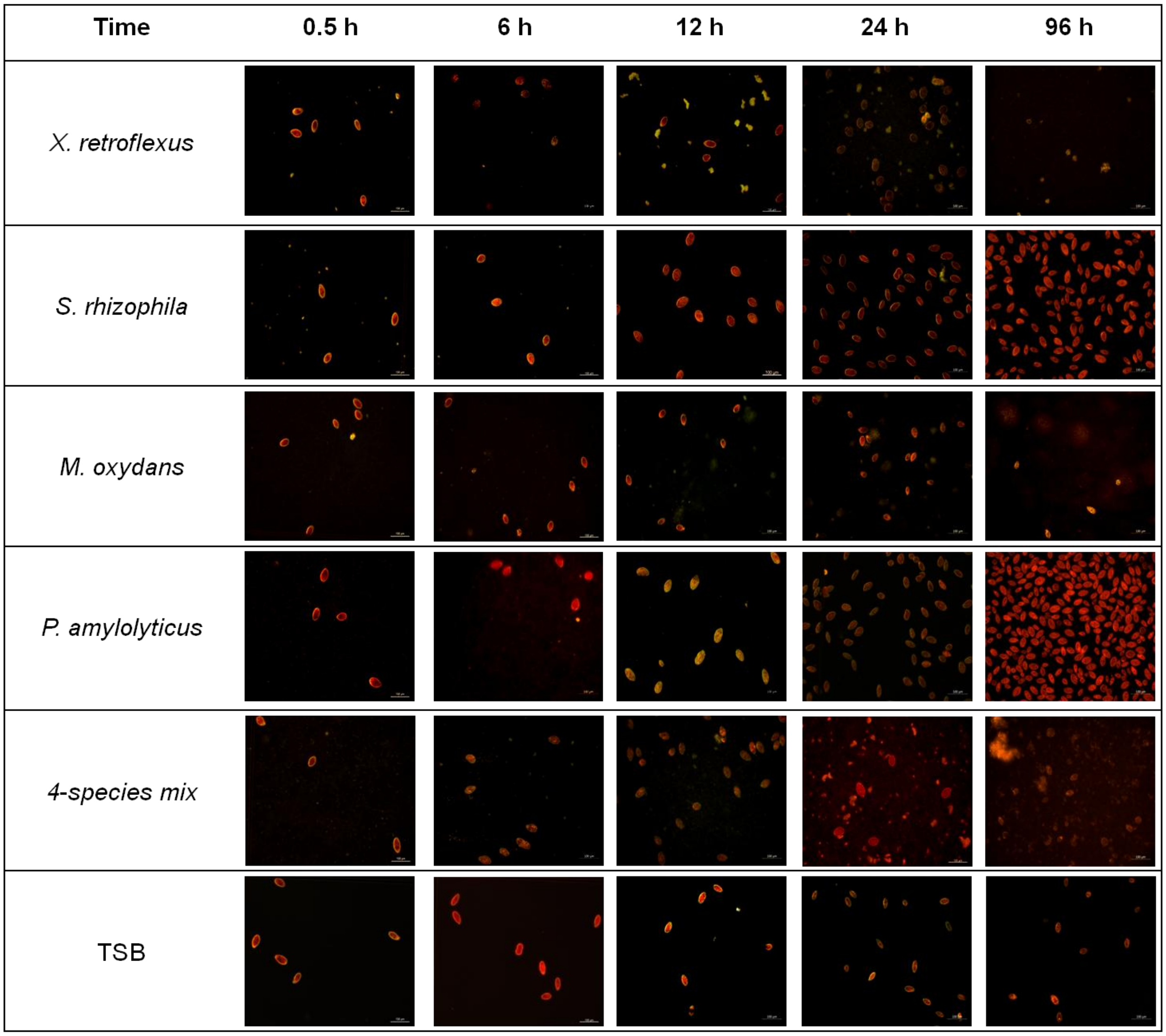

Supplement: Supplementary file 1 [file Image_1.PDF]
